# Case Report: Changes of Vascular Reactivity and Arterial Stiffness in a Patient With Covid-19 Infection

**DOI:** 10.3389/fcvm.2021.671669

**Published:** 2021-05-12

**Authors:** Philipp Jud, Harald H. Kessler, Marianne Brodmann

**Affiliations:** ^1^Division of Angiology, Department of Internal Medicine, Medical University of Graz, Graz, Austria; ^2^Diagnostic and Research Institute of Hygiene, Microbiology, and Environmental Medicine, Medical University of Graz, Graz, Austria

**Keywords:** Covid-19, endothelial dysfunction, vascular reactivity, arterial stiffness, vasculopathy

## Abstract

Covid-19 infection may be associated with a higher incidence developing cardiovascular complications, however, the underlying mechanisms contributing to cardiovascular complications are largely unknown, while endothelial cell damage may be present. We want to report a 24-year-old woman with Covid-19 infection who had undergone measurements of vascular reactivity and arterial stiffness, including flow-mediated dilation (FMD), nitroglycerin-mediated dilation (NMD), aortic pulse wave velocity (PWV), augmentation index and carotid intima-media-thickness (cIMT) at the time when Covid-19 was diagnosed. Reduced FMD of 0.0% and NMD of 15.5% were observed, while PWV (5.9 m/s), Aix (27%) and cIMT with 0.4 mm of both common carotid arteries were unremarkable. Repeated measurements of FMD, NMD, PWV, Aix, and cIMT 6 weeks after Covid-19 infection revealed persistently reduced FMD (0.0%), while NMD (17.24%), PWV (5.6 m/s) and augmentation index (13%) ameliorated. This case suggests potential impact of Covid-19 infection on endothelial function, also in young Covid-19 patients without any co-morbidity.

## Introduction

Covid-19 is caused by the severe acute respiratory syndrome coronavirus 2 (SARS-CoV-2) affecting primarily the respiratory system. Patients with cardiovascular comorbidities have an increased risk of in-hospital death and Covid-19 infection may lead to a higher risk of cardiovascular complications like heart failure, venous thromboembolism or stroke (1–4). Although prior data suggested a direct viral infection of the endothelial cell and diffuse endothelial inflammation which may promote to cardiovascular changes in Covid-19, a recent study assumed that direct endothelial infection by SARS-CoV-2 *via* angiotensin-converting enzyme 2 (ACE2) receptors is unlikely as there is a lack of ACE2 in human endothelial cells (5, 6). Furthermore, other pathways have been suggested contributing also to endothelial changes in Covid-19 (7–9). We report a 24-year-old woman with Covid-19 infection who had undergone measurements of vascular reactivity and arterial stiffness on the day of proven Covid-19 infection and 6 weeks after infection.

## Case Report

A 24-year-old woman underwent measurements of flow-mediated dilation (FMD), nitroglycerin-mediated dilation (NMD), pulse wave velocity (PWV), and carotid intima-media-thickness (cIMT) due to a preventive medical check-up at the beginning of December 2020. She was otherwise healthy, had a body-mass-index of 23.8 kg/m^2^ without any known atherosclerotic risk factor and worked as a secretary for a medical office in a hospital. Additionally, she was a non-smoker without a family history of cardiovascular disease and did not take any medications. Due to governmental initiated shutdown in Austria from November 3rd, 2020 to December 23rd, 2020, the patient refrained from sports activities during that shutdown, but she was active with regular sport activity of 30 min three times a week prior to that shutdown.

Measurements of FMD, NMD, cIMT, and pulse-wave analysis were performed in the morning between 7:00 a.m. and 9:00 a.m. after overnight fasting in a temperature-controlled (22–24°C) and quiet room by one trained technician. At the beginning of the FMD measurement, a blood pressure cuff was placed on the right forearm below the antecubital fossa and the baseline diameter of the right brachial artery was examined in a longitudinal plane between 2 and 7 centimeters proximal to the antecubital fossa in the patient. Three end-diastolic diameters between two intimal layers were measured ECG-gated during image acquisition in a one-centimeter-long segment of the brachial artery. Subsequently, the cuff was inflated >50 mmHg above the resting systolic pressure for 5 min, then deflated and 60 s after cuff release, the post-ischemic diameter of the brachial artery was measured. During a rest of 15 min, pulse-wave analysis including measurement of the aortic PWV and augmentation index was performed on the left arm and calculated *via* the oscillometric device Mobil-O-Graph® (I.E.M. Mobil-O-Graph, I.E.M., Cockerillstr., Stolberg, Germany) by an automated analysis. A size-adjusted cuff was placed on the patient's left upper arm about 2-4 centimeters above the antecubital fossa in supine position and subsequent pulse-wave analysis was performed, while the patient did not to speak or move over the whole pulse-wave analysis. Also, during the same rest of 15 min, the patient underwent measurement of the cIMT of both common carotid artery in supine position using a high-resolution linear array probe with 8–13 MHz (Siemens ACUSON S2000™, Siemens Healthcare Corp., Henkelstr., Erlangen, Germany). The thickness of the intimal and medial layers of the common carotid wall was measured on frozen longitudinal images in at least one-centimeter-long segment of the artery. After that rest of 15 min, the diameter of the right brachial artery was recorded similar to the technique described for FMD before and 5 min after sublingual administration of 0.4 mg glyceryl trinitrate spray. FMD and NMD measurements were performed with an 8–13 MHz linear array transducer using a conventional ultrasound scanner (Siemens ACUSON S2000™, Siemens Healthcare Corp., Henkelstr., Erlangen, Germany). Most recommendations for the measurement of FMD and NMD were fulfilled according to recent guidelines (10). The measurements revealed a reduced FMD of 0.0% and a reduced NMD of 15.5% according to proposed reference values (11). Pulse-wave analysis revealed a PWV of 5.9 m/s and an augmentation index of 27% while ultrasonography revealed a cIMT of 0.4 mm of both common carotid arteries.

**Figure 1:**
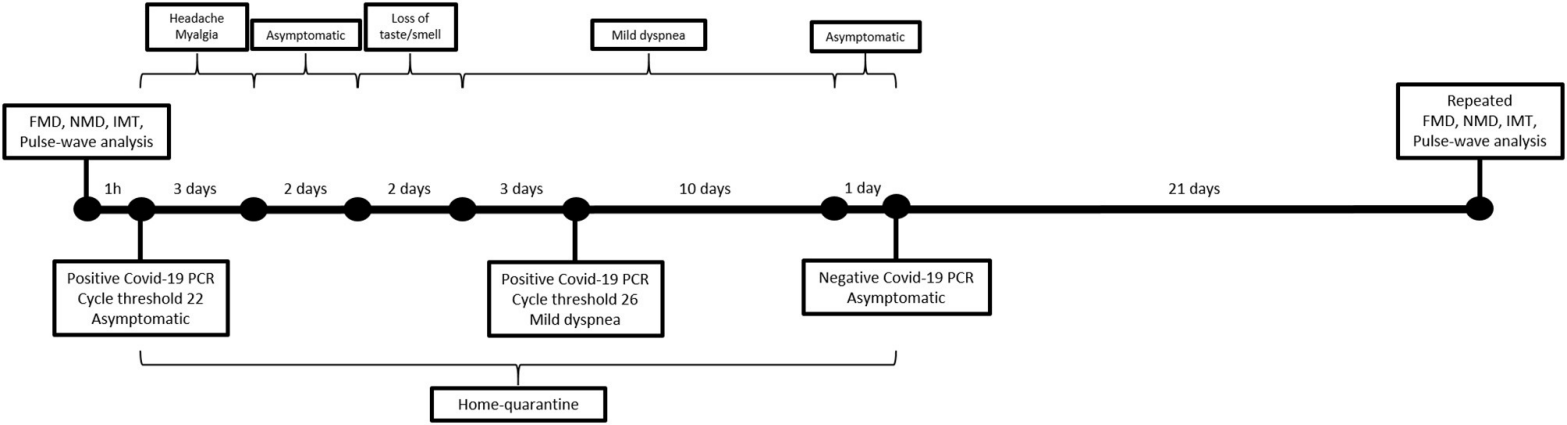
Timeline of patient's Covid-19 infection with symptoms and performance of laboratory and clinical measurements.

The patient was asymptomatic at the time of the respective measurements without potential symptoms of Covid-19 infection or any other infection. One hour after the respective measurements, testing for Covid-19 by polymerase chain reaction (PCR) was performed in that patient due to a routine testing for hospital staff which confirmed an acute Covid-19 infection with a cycle threshold of 22. The initial physical examination including auscultation was unremarkable with a body temperature of 36.6°C and a blood pressure of 127/88 mmHg. Measurement of oxygen level and chest x-ray were not performed as the patient was asymptomatic without respiratory symptoms. There was only a slightly elevated C-reactive protein (8.4 mg/L, reference value 0–5 mg/L) without lymphopenia and lipid parameters were also normal. The patient was subsequently home-isolated and was advised to monitor her health. During home-quarantine, the patient developed headache and myalgia within the first 3 days, which were treated by acetaminophen on demand and resolved afterwards, followed by loss of taste and smell as well as by mild dyspnea on exertion after the fifth and seventh day of home quarantine, respectively. On the tenth day of quarantine, Covid-19 PCR was performed again with a cycle threshold of 26. The patient was asymptomatic after 20 days of initial Covid-19 PCR and repeated PCR testing for Covid-19 was negative on the 21st day after initial Covid-19 PCR. A timeline of patient's Covid-19 infection with symptoms and performance of Covid-19 PCR and clinical measurements are shown in Figure 1.

Six weeks after initial Covid-19 testing, measurements for FMD, NMD, cIMT, and pulse-wave analysis were repeated by the same measurement methods as describes above evaluating changes of the respective parameters. FMD remained unchanged with 0.0% while NMD ameliorated to 17.24%. Furthermore, also PWV with 5.6% and augmentation index with 13% decreased while cIMT was unchanged.

## Discussion

We demonstrated with our case a potential impact of Covid-19 infection on endothelial dysfunction. Prior investigations of endothelial changes in Covid-19 infection have demonstrated direct viral infection of endothelial cells and endothelial inflammation with microthrombi and microangiopathy (5, 12). As the vascular endothelium is essential for the maintenance of vascular homoeostasis, dysfunction of the endothelium may result in cardiovascular changes. So far, there are only limited data about the pathophysiological mechanisms how SARS-CoV-2 contributes to endothelial dysfunction. While potential interactions of SARS-CoV-2 with ACE2 receptors have been suggested initially, recent data indicate that there is lacking evidence of ACE2 receptors expression on human endothelial cells assuming thus that direct infection of endothelial cells by SARS-CoV-2 is unlikely (5, 6, 13). Besides potential microvascular damage, also macrovascular damage may be promoted by Covid-19 infection since low values of FMD and NMD of the brachial artery were present in our patient. Additionally, amelioration of NMD, aortic PWV and Aix were observed after Covid-19 infection which indicates that Covid-19 infection may influence vascular homeostasis also in large arteries. Brachial FMD and NMD as well as aortic PWV are proven predictors of cardiovascular events and mortality and changes of those parameters are also associated cardiovascular events and mortality (14, 15). So far, data evaluating vascular reactivity or arterial stiffness in Covid-19 infection are very limited. Only one study investigated FMD and PWV in young adults 4 weeks after positive testing for SARS-CoV-2 revealing significantly lower values of FMD and higher values of PWV in the group of subjects with a suffered SARS-CoV-2 (16). However, data about vascular reactivity and arterial stiffness in acute Covid-19 infection are still lacking and follow-up changes of these parameters during a Covid-19 infection have not been investigated yet.

Underlying pathways by which SARS-CoV-2 may contribute to endothelial dysfunction are yet unknown. Our case and previous data suggest that both, direct cytotoxicity and indirect endothelial injury promote to endothelial dysfunction. Besides a potential but unlikely pathway of SARS-CoV-2 with ACE2 receptors, other pathways promoted by inflammatory mediators including interleukin-6 and prothrombotic mediators, like von Willebrand factor and neutrophil extracellular traps, may result in widespread inflammation and also in endothelial dysfunction (5–8, 13, 17, 18). As acetaminophen has only a weak anti-inflammatory effect, potential interaction of acetaminophen on inflammatory mediators which may affect endothelial dysfunction can be excluded (19). Additionally, as FMD and NMD indicates bioavailability of nitric oxide and PWV and augmentation index are parameters of arterial elasticity, we hypothesize that SARS-CoV-2 exhibits also an influence on nitric oxide metabolism and morphological changes of the arterial wall.

One limitation of our measurements was that we did not fulfill all recent recommendations for the assessment of FMD and NMD according to recent guidelines (10). Recommendations regarding subject preparation, operator-dependent factors and protocol were fulfilled, except for the recommended dose of sublingual glyceryl trinitrate. In our case, 0.4 mg glyceryl trinitrate was used instead of recommended 25 μg glyceryl trinitrate. Additionally, all other recommendations for technique and analysis were fulfilled, except for continuous measurement of velocity and diameter using simultaneous live duplex ultrasound, the use of continuous edge-detection and wall tracking software and calculating peak diameter and shear rate stimulus, since such a software was not available. Instead, offline analysis by a blinded observer was performed. Other limitations are that we conducted measurements of vascular reactivity and arterial stiffness only in one patient with Covid-19 infection and the lacking comparison of the results to a potential healthy, sex- and age-matched control subject.

Our case demonstrated that endothelial dysfunction may be present at a very early stage of Covid-19 infection and seems to be partly persistent even if SARS-CoV-2 is not detectable anymore. Our patient was asymptomatic at the time of verified Covid-19 infection when measurements of vascular reactivity and arterial stiffness were performed and symptoms occurred a few days later. It needs to be elucidated if parameters differ between asymptomatic and symptomatic patients as well as between patients with a different severity of symptoms. Furthermore, it needs to be elucidated if parameters of vascular reactivity and arterial stiffness remain altered as a long-term consequence of Covid-19 or if these changes may be present only in the acute phase of this infection. Moreover, studies evaluating parameters of vascular reactivity and arterial stiffness as potential predictors for cardiovascular events and mortality need to be performed.

In conclusion, we could demonstrate that infection by SARS-CoV-2 may alter different parameters of vascular reactivity and arterial stiffness probably by causing direct and indirectly endothelial dysfunction, which may promote to cardiovascular complications in patients with Covid-19 infection. Further studies evaluating parameters of endothelial dysfunction are urgently needed.

## Data Availability Statement

The datasets presented in this article are not readily available because all data are listed within the article. Requests to access the datasets should be directed to philipp.jud@medunigraz.at.

## Ethics Statement

Ethical review and approval was not required for the study on human participants in accordance with the local legislation and institutional requirements. The patients/participants provided their written informed consent to participate in this study. Written informed consent was obtained from the individual(s) for the publication of any potentially identifiable images or data included in this article.

## Author Contributions

PJ and MB contributed to conception and design of the study. HK contributed to data analysis. PJ wrote the first draft of the manuscript. All authors contributed to manuscript revision.

## Conflict of Interest

The authors declare that the research was conducted in the absence of any commercial or financial relationships that could be construed as a potential conflict of interest.
